# Influenza and respiratory syncytial viruses: Efficacy of different diagnostic assays

**DOI:** 10.12669/pjms.314.7003

**Published:** 2015

**Authors:** M.M. Rahman, K.K. Wong, H. Alfizah, S. Hussin, I. Isahak

**Affiliations:** 1 M. M. Rahman, Department of Medical Microbiology & Immunology, Faculty of Medicine, The National University of Malaysia, Cheras, 56000, Kuala Lumpur, Malaysia; 2 K.K. Wong, Department of Medical Microbiology & Immunology, Faculty of Medicine, The National University of Malaysia, Cheras, 56000, Kuala Lumpur, Malaysia; 3 H. Alfizah, Department of Medical Microbiology & Immunology, Faculty of Medicine, The National University of Malaysia, Cheras, 56000, Kuala Lumpur, Malaysia; 4 S. Hussin, Department of Medical Microbiology & Immunology, Faculty of Medicine, The National University of Malaysia, Cheras, 56000, Kuala Lumpur, Malaysia; 5 I. Isahak, Department of Medical Sciences, Faculty of Medicine and Health Sciences, Universiti Sains Islam Malaysia (USIM), 55100 Kuala Lumpur, Malaysia

**Keywords:** Influenza virus, Respiratory Syncytial Virus, Cell culture, Immunoflurescence Assay, Real-time polymerase chain reaction (rRT-PCR)

## Abstract

**Objective:**

To determine the efficacy of cell culture, immunoflourescence Assay (IFA) and real time polymerase chain reaction (rRT-PCR) in relation to diagnosis of influenza and Respiratory Syncytial Virus (RSV).

**Methods:**

Total 2781 specimens of throat swabs and nasopharyngeal aspirates were obtained from patients suspected of respiratory viruses’ infections from January 2009 to December 2011 at Universiti Kebangsaan Malaysia Medical Centre(UKMMC). The specimens were processed by cell culture and immunoflurescence assay (IFA) and (rRT-PCR).

**Results:**

Thirty three (1.19%) specimens were positive for influenza virus A and 42 (1.51%) were positive for RSV by cell culture and IFA. On the other hand, rRT-PCR was able to identify 189 of 505 (37.43%) specimens in which 65 were influenza A virus and 124 were RSV. Sensitivity of rRT-PCR was 100% for both influenza A virus and RSV and specificity was 88% and 77% for influenza A virus and RSV, respectively.

**Conclusion:**

rRT-PCR diagnosed respiratory viruses in shorter time with a high level of sensitivity in comparison to conventional assays - cell culture and IFA. These advantages help in managing patients by saving cost and hospitalization stay.

## INTRODUCTION

Respiratory viruses are the major causes of respiratory illness throughout the world.^1^ Among these viruses influenza and respiratory syncytial virus are predominant.^2^ These viruses cause morbidity and mortality of young children, elderly and immuno-compromised patients. The most common clinical manifestations of these viral infections range from fever, sore throat, and myalgia to more serious complications including bronchitis, pneumonia and death.^3^

Three known types of influenza viruses (A, B, and C) currently circulate in the human population, Types A and B are associated with clinically important respiratory illness.^4^ Respiratory syncytial virus (RSV) is best known for its tendency to cause bronchiolitis in infants, but it can infect all age groups causing upper and lower respiratory tract infections ranging in severity from subclinical infections to pneumonia and death.^5^

Each year, influenza viruses cause illness in millions of cases associated with various respiratory syndromes and approximately 500,000 deaths.^6^ Globally, about 20% of children and 5% of adults develop symptomatic influenza each year.^7^ Similarly RSV is associated with 40–90% of bronchiolitis cases in children less than 5 years of age and 50% of pneumonia cases in children less than 2 years of age.^8^ Therefore, respiratory virus infections represent a major public health problem because of their worldwide occurrence, ease of spread in the community and considerable morbidity and mortality. New respiratory viruses with epidemic and pandemic potential continue due to their genomic nature.^9^

In Malaysia, the Institute for Medical Research (IMR) of Kuala Lumpur screened respiratory illness on 7,117 respiratory specimens during 2005-2009 and reported for the identification of influenza viruses in 17.3% in 2005, 31.6% in 2006, 12.8% in 2007, 10.2% in 2008 and 13.5% in 2009.^10^

Quick detection of viral agent provides guidance for the prompt management of the patients showing respiratory illness. Therefore, the present study was undertaken to determine the efficacy of cell culture, immunofluoescent assay (IFA) and real time Polymerase hain eaction (rRT-PCR) in relation to diagnosis of influenza and RSV.

## METHODS

### Study population

The study was undertaken at Universiti Kebangsaan Malaysia Medical Centre (UKMMC) from January 2009 to December 2011. A total of 2781 throat swabs and respiratory aspirates were collected in Viral Transport Medium (VTM) during the period in cool condition. These were sent to the laboratory of Medical Microbiology and Immunology, UKMMC for virus isolation and identification.

### Patients’ information

Patients of all ages, gender and ethnic groups suffering from respiratory illness were recorded from the patients’ information sheet provided with the specimens by the clinicians.

### Ethical approval

The study protocol was approved by UKMMC Ethical Committee (FF-320-2011).

### Propagation of viruses in cell culture

Madin-Darby Canine Kidney (MDCK) cells (ATCC number, CCL-34^TM^) and HEp-2 cells (ATCC number, CCL-23^TM^) were purchased from ATCC (Manassas, VA 20110, USA.) and used for the propagation and initial detection of viruses based on cytopathic effect. Eagle Minimal Essential Medium (EMEM) (Gibco, USA) supplemented with 10% Fetal Bovine Serum (FBS) (Gibco, USA) were used to grow the MDCK and HEp-2 cell lines. These were used in combination to observe the comparative efficacy for the growth of both influenza and RSV. Method of Ken et al.^11^ was followed to set up of assay.

### Indirect Immunoflourescence Assay

The presence of a specific virus was confirmed by indirect immunoflourescence staining. The Light Diagnostics™ Respiratory Panel 1 Viral Screening and Identification Kit (Millipore, USA) was used for the qualitative confirmation of influenza and RSV. Briefly, acetone fixed slide were stained with Respiratory Virus and specific identification antibody and FITC-labelled Anti-Mouse IgG Conjugate were added on it. The slides were then examined using a fluorescence microscope (UV Microscope, Olympus, Japan, BX50). The method of Ken *et al*.^11^ was also followed in this regard.

### Molecular detection of influenza virus and RSV

The specific nucleotide for the primer and probe in the gene coding from the complete genome of RSV and influenza viruses were obtained from gene bank. Molecular assay was carried out for the identification of both the viruses as per the procedure Ken et al.^11^

## RESULTS

### Identification Influenza A and RSV only based on IFA

**Fig. 1:**
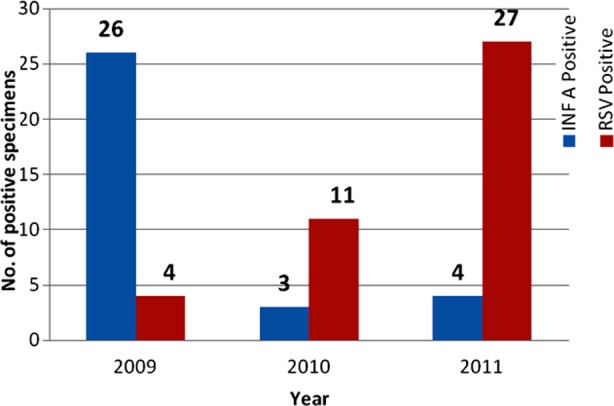
Positive influenza A and RSV by IFA during 2009 -2011.

A total of 2781 specimens were processed during study period and it was observed that 33 (1.19%) were positive for influenza A and 42 (1.51%) positive for RSV (Fig.1). In 2009, four cases of RSV and 26 cases of influenza A virus in 2010, 11 strains of RSV and 3 strains of influenza A virus and in 2011, 27 cases of RSV and 4 cases of influenza A were isolated. The average isolation rate per year was 1.51% for RSV and 1.18% for influenza A virus.

### Comparative sensitivity of influenza A and RSV virus isolation in 3 different cell lines

**Fig. 2:**
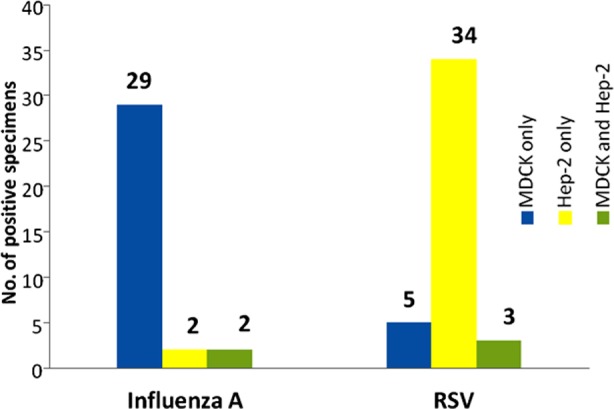
Positive influenza A virus and RSV by MDCK and HEp-2 cells.

MDCK, HEp-2 and MDCK and HEp-2 were used in combination to determine the sensitivity for the propagation of both influenza and RSV and production of characteristics CPE. It revealed (Fig.2) that 93% of influenza A viruses propagated efficiently in MDCK cells and 81% of RSV propagated in HEp-2 cells. However, 3 strains of influenza A and 2 strains of RSV propagated only in combined MDCK and HEp-2 cell lines. In this regard we observed that selection of cell lines in propagating identifying viruses plays an important role.

### Comparative efficacy of Immunoflourescence assay and rRT-PCR

A total of 505 specimens were tested for the identification of both influenza and RSV and it was observed that only 12 cases were positive by immunoflourescence assay; 3 were influenza A virus and 9 were RSV.

**Fig. 3:**
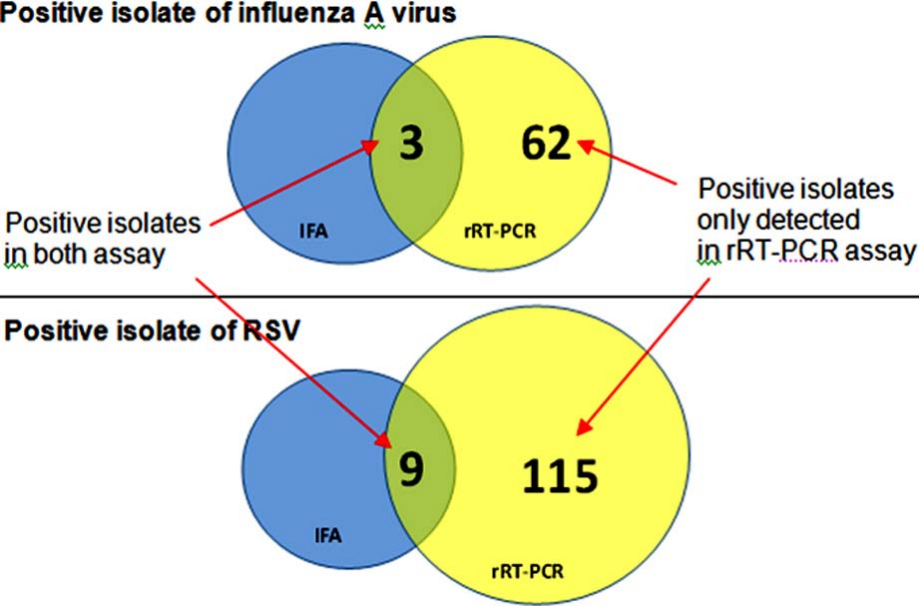
Number of positive isolates of influenza A virus and RSV detected by cell culture and Immunofluorescence Assay and rRT-PCR.

The 12 specimens’ positive for influenza A virus and RSV by conventional virus isolation technique were shown to be positive by rRT-PCR assay too. In addition, rRT-PCR detected positive an additional 177 specimens that included 115 cases of RSV and 62 cases of influenza A virus. The positivity of rRT-PCR assay for influenza A virus was 12.9% (65/505) and for RSV it was 24.6% (124/505). It was proved that rRT-PCR is more efficient than conventional method of virus isolation (Fig.3) Sensitivity of the in-house developed rRT-PCR was 100% for both influenza A virus and RSV and specificity was 88% and 77% for influenza A virus and RSV, respectively.

## DISCUSSION

Comparative efficacy study showed that positivity of rRT-PCR assay for influenza A virus was 12.9% (65/505) and RSV was 24.6% (124/505). Therefore it is further established that molecular methods yielded efficient detection of clinical specimens than conventional methods.(Fig.3) Sensitivity of the in-house developed rRT-PCR was 100% for both influenza A virus and RSV and specificity was 88% and 77% for influenza A virus and RSV, respectively.

In the study we were unable to isolate influenza B virus for study period. Reports showed that only few studies proved high sensitivity for RSV and influenza virus detection when cell culture was performed with fresh samples immediately after collection from the patients.^12^ It was observed that there are a few critical factors that might contribute to the low detection rate of virus isolation assay in this study. Firstly, transportation of specimens from ward to laboratory using hospital postage system led to delays along the way. This affected the viability of viruses in the specimens as proved by a study showing lesser percentage of positive samples by virus culture. It is probably due to viral inactivation during transport where viability is essential for propagation and detection.^13^

Some authors in this regards mentioned that virus detection with cell culture and subsequently by IFA assay rarely provide quick results to impact treatment, however, it does provide important confirmation of clinical diagnosis.^14^ In the study, MDCK and HEp-2 cell lines were used for virus isolation due to their high susceptibility of infection with various influenza virus strains and RSV strains. In this study, 93% of influenza A virus were isolated from MDCK cell and 81% of RSV isolated from HEp-2 cell only. However, 3 strains of influenza A and 2 strains of RSV propagated only in combined MDCK and HEp-2 cell lines. In this regard we observed that selection of cell lines in propagating and identifying viruses plays an important role. Our data support a previous study which showed that both MDCK cells and HEp-2 cells were capable of efficient virus propagation.^15^ MDCK cell line is the most widely used and is highly permissive for propagation of influenza virus. It displays better sensitivity compared with other cell lines.^16^ Our data suggest that both MDCK cell line and HEp-2 cell line could be used for influenza virus and RSV isolation. MDCK cell line is suitable for influenza whereas HEp-2 cell line is suitable for RSV.

In this study, molecular assay rRT-PCR successfully detected 124 specimens of RSV and 65 specimens of influenza A virus with sensitivity of 100% for both influenza A virus and RSV and specificity of 88% and 77% for influenza A and RSV, respectively. All specimens positive by virus culture and IFA were also positive by rRT-PCR. This result is consistent with the other study that detected respiratory viruses easily within shortest possible time with high level of sensitivity.^17^ A study showed 23% of positive samples were missed by viral culture, which was most likely due to the amount and viability of the viruses present in the clinical samples.^18^ Rapid diagnosis of RSV and influenza is useful for children, elderly or hospitalized patients, so that appropriate infection control measures might be taken and inappropriate antibiotic use could be limited. Finally, molecular assay rRT-PCR was further proved to be a better tool in comparison to conventional assays -cell culture and IFA for the detection of respiratory viruses in shorter time so that prompt management of the patients could be done saving cost of medication and hospitalization stay.
